# Analysis of the Importance of Oxides and Clays in Cd, Cr, Cu, Ni, Pb and Zn Adsorption and Retention with Regression Trees

**DOI:** 10.1371/journal.pone.0168523

**Published:** 2017-01-10

**Authors:** Juan José González-Costa, Manuel Joaquín Reigosa, José María Matías, Emma Fernández-Covelo

**Affiliations:** 1 Department of Plant Biology and Soil Science, Universidad de Vigo. Vigo, Spain; 2 Department of Statistics and Operational Research, Universidad de Vigo. Vigo, Spain; University of Delhi, INDIA

## Abstract

This study determines the influence of the different soil components and of the cation-exchange capacity on the adsorption and retention of different heavy metals: cadmium, chromium, copper, nickel, lead and zinc. In order to do so, regression models were created through decision trees and the importance of soil components was assessed. Used variables were: humified organic matter, specific cation-exchange capacity, percentages of sand and silt, proportions of Mn, Fe and Al oxides and hematite, and the proportion of quartz, plagioclase and mica, and the proportions of the different clays: kaolinite, vermiculite, gibbsite and chlorite. The most important components in the obtained models were vermiculite and gibbsite, especially for the adsorption of cadmium and zinc, while clays were less relevant. Oxides are less important than clays, especially for the adsorption of chromium and lead and the retention of chromium, copper and lead.

## Introduction

### Rationale and background

Heavy metal contamination id defined as excessive contamination from a series of metal elements, most of which are potentially toxic for the organism in a given ecosystem [1]. As for the formal definition of these elements, there is not a clear criterion, and there are numerous definitions and come classifications [2].

Although contamination by these elements affects all the levels in an ecosystem [3,4], the microorganisms in the soil are the first to be harmed, from bacteria to protozoa. That is why these organism show differences in their adaptation to said contamination [5].

Within these adaptations, bacteria are the group showing the greater variability, given that the number of possible responses depends on their metabolic complexity and ranges from passive adsorption in the cell wall from active expulsion of heavy metals with energy waste [6]. Some of these mechanisms, such as passive adsorption in the wall cell, also happen in higher microorganism, such as heavy-metal-tolerant fungus (*Aspergillus niger* and *Penicilium sp* tolerating Cr and Cd [7]. In other cases, they use other mechanisms, such as certain modified fatty acids, which gives them the capacity to resist high concentrations of heavy metals from industrial waste, for example [8]. Lastly, in the case of protozoa, they have different mechanisms to repel and resist heavy metal, depending on whether they are autotrophic or heterotrophic species. Protozoa of genus *Euglena*, such as species *Euglena gracilis*, show both types of nourishing. It is in these cases where it can be observed that heterotrophic stages or species show detoxification methods based on glutathione, while autotrophic species or stages show metal expulsion mechanisms similar to those found in plants, such as phytochelatins synthesis (phytochelatins being the substances that accumulate heavy metals, preventing them from exert their toxic action) [9, 10].

In plants, heavy metals and other trace metals, such as aluminium, can affect their photosynthetic efficiency and vegetative growth, making it slower and less vigorous and triggering premature ageing [11, 12].

For this reason, plants also have defense mechanism against heavy metals, such as passive adsorption [13] or the productions of heavy-metal chelating substances (phytochelatins) [14]. Cotransport with other ions, such as sodium, also exists, and in some cases there may be resistance to certain metals, such as Ni, but hypersensitivity to others, such as Pb [15]. Lastly, in some cases there are changes to the expression of the whole genome, there being in some cases specific genes that are expressed only at the moment when particular metal (e.g. aluminium) is found in the soil [16].

So far in this paper, we have discussed mainly the dynamics of cadmium, chromium and nickel. Other heavy metals (included in this study) show the following effects:

- Copper affects mostly plants’ leaves. It is very abundant in nature, both in its elemental form and as minerals and salts, being a component of certain rocks, such as serpentinites [17].
- Nickel is found in various types of rocks and salts, and it is a widely use mineral in metallurgy. It is also a micronutrient for plants, although it is toxic for them in much lower concentrations than it is for animals and humans [18].
- The majority of the lead present in the soils comes from anthropogenic emissions, especially when organic additives containing this metal were used in petrol, although it is also naturally found in certain types of soils. This material is toxic for humans, especially for children, and comes from various sources such as dust, water, air or food. As for plants, it is accumulated especially in the roots [19].
- Zinc is an essential metal for both plants and animals, but it can be harmful for both in high concentrations [18, 20].

Heavy metals can concentrate in the food chain (soil to plant and plant to consumer, directly or through animals) thus becoming a threat to human health [21].

For all these reasons, decontamination methods are being studied and implemented, including physical methods like mechanical separation [22], electrokinetic remediation [23], chemical washing (ex-situ soil washing or in-situ soil flushing), soil amendments, including lime or other natural materials, chelating agents, nanomaterials or biological products [24, 25].

Other methods to decontaminate soils which are contaminated by heavy metals are those called remediation, which use the microorganisms in the soil to decontaminate it [26] or phytoremediation, that is the use of plants to extract the heavy metals, including the use of hyperaccumulator plants, and the different processes called phytostabilisation and phytoextraction [27, 28]. These methods do not have the same environmental impacts as the others. Finally, active research in the use of transgenic plants especially tailored to extract heavy metals is producing interesting results [29].

Nowadays, new analysis methods to prevent contamination by heavy metals are being researched, especially for agricultural practices, such as soil fertilization [30], but this is also gaining strength in other fields, such as decorative plants [31].

Nonetheless, the soil is the place where the behaviour of heavy metals in an ecosystem at the bioavailability level can be predicted. The different mineral and organic components of the soil can be a barrier for heavy metals by means of the adsorption and retention mechanisms [17, 32].

### Aim of the study and source of the data

The aim of this study is to Rank the different components of the soil according to their relevance coefficient in a regression tree model, remarkably oxides and clays.

As for the source of the data used, they were obtained from different soils in Galicia (NW Spain), including samples of soils which, due to their composition, are naturally contaminated by heavy metals, given that high concentrations of certain metals can interfere in the adsorption and retention of others [33].

## Materials and Methods

### Characteristics of the sample

The data analysed in this study were gathered by Covelo EF for her thesis [17] from 14 soils with three samples each (42 samples). These soils characteristics are listed in Table 1. No permission was needed for sampling in those locations.

**Table 1:** Features of the analysed soils according to the World Reference [34] and the type of bedrock where they lie.

No.	Name	Coordinates	Type	Bedrock
1	UH—1	42° 11’ 20” N 8° 39’ 56.2” W	Humic Umbrisol	Schist
2	UH—2	42° 11’ 5” N 8° 40’ 36.2” W	Humic Umbrisol	Gneiss
3	UH—3	42° 11’ 24” N 8° 41’ 01” W	Humic Umbrisol	Gneiss
4	UH—4	42° 12’ 30” N 8° 41’ 0.31” W	Humic Umbrisol	Gneiss
5	RD	42° 5’ 47” N 8° 37’ 30.1” W	Dystric Regosol on Umbric Acrisol	Quaternary sediments
6	AU	42° 5’ 54” N 8° 37’ 31.7” W	Umbric Acrisol	Quaternary sediments
7	RE	42° 1’ 00” N 8° 40’ 55” W	Eutric Regosol	Quartz and granite
8	AH	41° 57’ 15” N 8° 45’ 59.1” W	Hortic Anthrosol	Quaternary sediments
9	AP	41° 56’ 21” N 8° 47’ 25.3” W	Plaggic Anthrosol	Slate, schist and paragneiss
10	FT	42° 06’ 52”N 8° 49’ 25.2” W	Thionic Fluvisol	Granite
11	CF	42° 54’ 48.9” N 8° 1’ 47.9” W	Ferralic Cambisol	Serpentine
12	PH	42° 48’ 34.4” N 8° 26’ 47.7” W	Haplic Podzol	Quartzite
13	13 LM	42° 57’ 47.7” N 7° 57’ 9.5” W	Mollic Leptosol	Serpentine
14	HF	42° 14' 32.6'' N 5° 34´ 21.7'' W	Fibric Histosol	Granite

In all of them, the following tests were performed, according to the protocols by [35].

- Determining the soil organic matter through wet assessment with potassium dichromate.
- Determining the soil texture by separating the different soil components (sand, clay, etc.) through wet and dry sieving.
- Determining the mineral identification and properties through techniques such as X-ray diffraction.
- Determining the soil exchange capacity.

As a result of these analyses, the following variables were determined Table 2.

**Table 2:** Description of the variables used.

Variable	Definition	Min. val.	Max. val.
**Humified organic matter**	Proportion of the humified fraction of the soil for each kg of dry soil	1.06 mg/Kg	**71.91 mg/kg**
**Sand percentage**	Proportion of fractions with a size over 50 micrometres in 100 grams of soil	26.71%	**75.82%**
**Silt percentage**	Proportion of fractions with a size between 30 and 50 micrometres in 100 grams of soil	1.67%	**34.56**
**Specific cation-exchange capacity**	Easiness with which one cation is replaced by other in the exchange positions of the soil components.	0.36	**11.22**
**Quartz content**	Proportion of quartz (silica) in 100 grams of dry soil	0.84%	**9.5%**
**Plagioclase content**	Proportion of plagioclase in 100 grams of dry soil	0%	**7.5%**
**Mica content**	Proportion of mica in 100 grams of dry soil	0%	**2.5%**
**Kaolinite content**	Proportion of kaolinite in 100 grams of dry soil	0%	**89%**
**Vermiculite content**	Proportion of vermiculite in 100 grams of dry soil	0%	**5.26%**
**Gibbsite content**	Proportion of gibbsite in 100 grams of dry soil	0%	**89%**
**Chlorite content**	Proportion of chlorite in 100 grams of dry soil	0%	**29.75**
**Hematite content**	Proportion of hematite (crystallized form of Fe oxide) in 100 grams of dry soil	0%	**33%**
**Mn oxides content**	Proportion of Mn oxides in milligrams per kg of soil	0 mg/Kg	**1.52 mg/Kg**
**Fe oxides content**	Proportion of Fe oxides in milligrams per kg of soil	2.47 mg/Kg	**74.36 mg/Kg**
**Al oxides content**	Proportion of Al oxides in milligrams per kg of soil	3.26 ng/Kg	**98.21 Mg/Kg**
**Cd adsorption**	Difference in the concentration of cadmium added and eluted when through a sample of soil or other material.	9.930%	**29.973%**
**Cd retention**	Concentration of Cd in the acetate buffer solution after sample	2.74%	**30.45%**
**Cr adsorption**	Difference in the concentration when through a sample of soil or other material.	14.691%	**80.684%**
**Cr retention**	Concentration of Cr in the acetate buffer solution after sample	11.768%	**77.005%**
**Cu adsorption**	Difference in the concentration of copper added and eluted when through a sample of soil or other material.	6.069%	**81.989%**
**Cu retention**	Concentration of copper in the acetate buffer solution after sample	2.341%	**78.725%**
**Ni adsorption**	Difference in the concentration of nickel added and eluted when through a sample of soil or other material.	3.601%	**33.576%**
**Ni retention**	Concentration of nickel in the acetate buffer solution after sample	1.688%	**28.693%**
**Pb adsorption**	Difference in the concentration of lead added and eluted when through a sample of soil or other material.	31.116%	**94.160%**
**Pb retention**	Concentration of lead in the acetate buffer solution after sample	26.617%	**89.516%**
**Zn adsorption**	Difference in the concentration of zinc added and eluted when through a sample of soil or other material.	2.921%	**31.021%**
**Zn retention**	**Concentration of zinc in the acetate buffer solution after sample**	**0.924%**	**29.365%**

As a result, the variables described in Table 2 were recorded together with their most relevant descriptive value. Certain components were observed to be absent from Galician soils, while the most adsorbed and retained metals are those which are competitively stronger [17].

Following these analyses, the adsorption isotherms for each soil and metal were drawn, and K_d_ or the distribution coefficient, and K_dΣ_, or the accumulated distribution coefficient, were calculated. These are defined as the quotient of the metal concentration in the soil in micromoles and that existing in the solution multiplied by 10^−3^. In the experiments where the K_d_ was determined, a metal solution with a concentration of 100 μmol L^-1^ was used, while the concentration for K_dΣ_ varied from 5 to 400 μmol L^-1^.

In this way, the adsorption and retention isotherms for the different metals are determined, and it is possible to work with these coefficients [17, 33, 36, 37, 38]. However, an analysis of the results showed they were irregular, and so these data were translated into the adsorption and retention percentages used in this study, which are referenced in Table 2. The comparison between the results obtained by her in [32, 33] with those obtained through a more accurate model validation and selection method is relevant.

At first, the possibility to consider Fe oxides and Al oxides as a single component was considered, given the existence of evidence regarding their colinearity [17, 33, 36, 37, 38], but the trees obtained were similar to those in [32, 33], so it was decided to treat them separately.

### Election of the statistical technique

In order to achieve the aforementioned aims, regression models were built showing the importance of the different soil components regarding the adsorption and retention capacity for the different heavy metals. Regression trees (CART) were used to built those models [40], mainly due to the fact that they are very flexible models, enabling to reproduce complex non-linear relationships between the independent variables (soil components) and the dependent variables (retention and adsorption). In this regard, linear models cannot be deemed, a priori, sufficient to capture the relationships between both groups of variables, and their use would imply unjustified restrictions to the form of said relationships.

The aforementioned advantage of CART is also shared by other machine learning techniques, such as neuronal networks or support vector machines [40], or other techniques that usually fall within non-parametric statistics, such as generalized additive models [41]. Nonetheless, trees have the following comparative advantages:

- They are very interpretable models, due to their graphic representation and to the fact that they can be described in terms of a natural language close to the researcher.
- Despite being non-linear models, where it is always difficult to analyse the influence of independent variables on the answer, regression trees allow us to perform and intuitive analysis of the importance of these variables from their behaviour during the process of building the tree.
- Their estimation (learning, in machine learning terminology) entails a perfectly assumable computational cost (the estimation of a model with a dozen independent variables and one dependant variable, using several hundreds of data, is performed in seconds), quite lower than that required by the other aforementioned techniques.

As a drawback, like any other machine learning or no-parametric techniques, their great adjustment capacity makes it necessary to control the complexity of the model in order to prevent data overfitting. In order to do that, model selection techniques are required, which were based on cross-validation methods in this study.

#### Regression trees

Regression trees (CART) are machine learning techniques to model a regression problem. They are of a non-parametric nature, for they do not postulate a priori a pre-fixed parametric model of the relationship between the explanatory variables and the answer, but rather adapt their structure to the relationship structure which is present in the data sample. In this regard, they are very flexible, since they can capture continuous non-linear relationships with a random complexity, but, for this same reason, they have a great capacity to adjust to the data, which makes it necessary to use techniques to control their complexity and prevent their natural tendency to overfit.

Given a data sample ${\left\{(x_{i},y_{i})\right\}}_{i=1}^{n}$ where $x_{i}\in X\subset R^{d}$ and $y_{i}\in Y\in R,\;i=1,..,n$, X is the input space and Y is the output space, the regression tree responds to the following model

$$\hat{y}(x)=\hat{f}(x)=\sum_{j=1}^{h}{\hat{c}}_{j}1_{A_{j}}(x)$$ (1)

Where ${\hat{c}}_{j}\in R$ and $1_{A_{j}}(x)$ is the indicative function assigning value 1 if *x*∈ *A*_*j*_ and zero otherwise. Thus, the regression tree splits the input space X into different regions *A*_*j*_ which are exhaustive and exclusive, and assigns the value ${\hat{c}}_{j}$ to all the individuals in each region *A*_*j*_.

The estimation problem of regression trees lies in determining the split ${\left\{A_{j}\right\}}_{j=1}^{h}$ (and the number *h* of regions), as well as coefficients ${\hat{c}}_{j}$ of the expression (1) above. In this study, this estimation has been carried out in the following stages:

- Estimation of the maximum-complexity tree
- Selection of the model.

Later, the importance of each of the variables was analysed. Each of these stages are briefly described below.

- **Estimation of the maximum-complexity tree:** The aim of this stage is to develop a maximum-complexity tree from which different lower-complexity trees are to be built. The most suitable from among these will be selected in the following model selection stage. The maximum complexity tree is built by making successive splits (branches) of the input space. Each split is defined by a condition of type *x*^*j*^ < *a* where *x*^*j*^ is one of the variables of the variable vector $x\in X\subset R^{d}$. The selection of the variable *x*^*j*^ and of the cut-off value *a* is done in such a way that the least squares criterion is minimized:
  $$(j,a)=\sum_{x^{j}<a}{\left(y_{i}-c_{1}\right)}^{2}+\sum_{x^{j}\geq =a}{\left(y_{i}-c_{2}\right)}^{2}$$ (2)
  where *c*_*k*_, *k* = 1, 2 is the average of the response variable in the individuals in each one of the two sets (leaves) of the splitting defined by *x*^*j*^ < *a*.
  The splitting process continues by applying the aforementioned method to each of the regions obtained up to that moment, although a series of stop criteria are reach which are aimed at avoiding excessively complex trees. A usual criterion is to have a lower limit to the number of individuals in each split (3 data in this study). Otherwise, the maximum tree would end up building a split which as many regions as individuals in the sample.
- **Selection of the model:** Once the maximum tree has been built, the different intermediate trees with decreasing complexity are built from it through the removal of sections (pruning). The optimal tree is selected from among these through an *m*-fold cross-validation method, as described below:
  1. The sample is split into *m* groups of a similar size.
  2. The maximum tree is built with the data from *m*-1 groups. Their prediction error is assessed in the data from the group that has been put aside, which have not been used by the algorithm.
  3. The number of sections producing a smaller quadratic prediction error in all the groups that have been being put aside from the estimation.
  4. A tree is estimated with this optimal number of sections, using all the data in the sample.
    Once the model has been estimated in step 4, its goodness of fit is assessed by calculating the coefficient of determination *R*^*2*^, by means of the expression:
    $$R^{2}=\frac{\sum_{i=1}^{n}{\left(\hat{f}(x_{i})-\bar{\hat{f}(x_{i})}\right)}^{2}}{\sum_{i=1}^{n}{\left(y_{i}-\bar{y}\right)}^{2}}$$ (3)
    where $\hat{f}(x_{i})$ is the prediction of the model for the i-th observation, i = 1,…,n.
- **Importance of the variables:** Each iteration of the successive splitting process implied in the estimation of the maximum-complexity tree results in the selection of the best variable to make the splitting. Nonetheless, it may be the case that other variables that are not selected for the different optimal splittings cold make good splittings (although not better than the chosen ones) and were never selected. For example, a variable which is always the second best for all the splittings could be the most important variable but would be "overshadowed" by those finally selected in the different splittings.

For this reason, a method to determine the importance of the different variables [39] is based on determining the sum of quadratic error decreases ΔR for each j-th variable that its use in each splitting t would yield, that is to say, the value ∑_t_ΔR(j, t), that is to say, the sum of all the benefits yielded by the use of the variable in each node.

All the process of estimation, selection of the model and assessment of the importance of its variables was carried out using R environment [42]. The estimation of the trees was performed by means of the package *Recursive splitting* (rpart), [43] and the assessment of the predictive capacity was developed and tailored in said environment.

## Results and Discussion

In this section, the trees obtained for the retention and adsorption of each of the heavy metals are shown, as well as the analysis of the importance of the soil features in each on said models.

In Table 3, the goodness of fit of the different models in terms of the coefficient of determination *R*^*2*^ obtained as described in the previous section is shown.

**Table 3:** Predictive capacity of the models built in terms of the R^2^ obtained in the cross-validation process.

	R^2^	R^2^ (Covelo)
Cdads	98.27	82.00
Cdret	95.26	85.40
Crads	98.30	95.74
Crret	98.37	95.84
Cuads	99.33	98.12
Curet	99.43	99.06
Niads	97.77	94.87
Niret	98.47	94.70
Pbads	99.83	99.27
Pbret	99.76	99.12
Znads	93.60	77.46
Znret	88.48	88.48

Considering the values of R2 included in Table 3, it can be established that the different goodness of fit values in the improved model are better than when considered separately, which means that, in every instance, the trees obtained are closer to the reality than those obtained by Covelo et al. [32, 33].

### Cadmium

The regression model for this metal works better for the adsorption process, with a goodness of fit of 98.27% of the total variation, while the percentage falls to 95.26% for retention.

**Fig 1:**
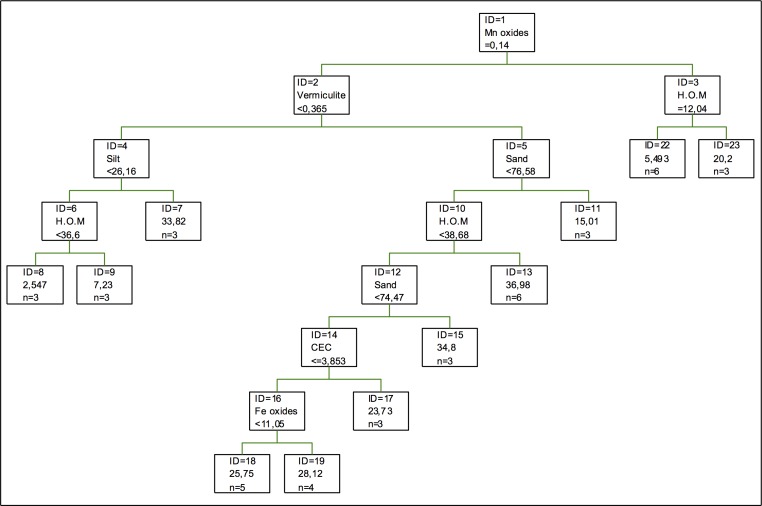
Regression tree for cadmium adsorption. The inequalities < or ≤ correspond to the left branches and the opposite (> or ≥) correspond to the right branches.

In the regression tree for cadmium adsorption, as it can be seen in Fig 1, the first splitting is done according to the Mn oxides, while vermiculite appears in the second splitting. However, the component appearing in three of the splittings is humified organic matter and the percentage of sand, the latter being less relevant. Despite humified organic matter having so much importance in the adsorption of cadmium, this metal is not one of the most adsorbed metals in soils with higher humified organic matter content a (humic umbrisols), which mainly adsorb chromium [44].

However, in the case of the coefficients of importance, the most important component for this model is the percentage of sand. As for clays and non-crystallized oxides, the most important ones are chlorite and Fe oxides, respectively. In the case of clays, chlorite is followed by kaolinite, gibbsite and vermiculite, the latter being the least important of all components. Although oxides, in this case, are not the most important components in the adsorption of this metal, their specific area makes them highly efficient in the adsorption process, especially Fe oxides, although this has to be in low concentrations, given that their adsorption capacity decreases with the increase in concentration [45].

**Fig 2:**
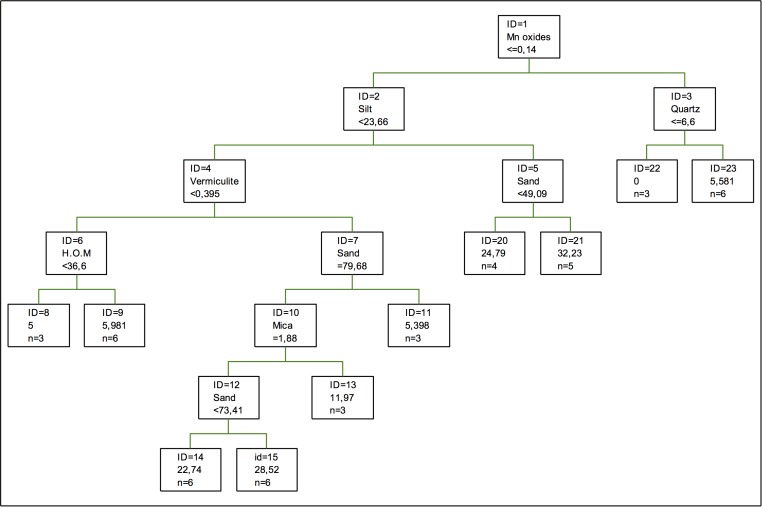
Regression tree for cadmium retention. The inequalities < or ≤ correspond to the left branches and the opposite (> or ≥) correspond to the right branches.

In the case of cadmium retention, as it can be seen in Fig 2, the first splitting is done again according to Mn oxides, followed in the second one by the proportion of quartz, silt and vermiculite and the percentage of sand. In general, this tree has a simpler structure than in the case of adsorption, so fewer components are needed for the regression [40]. On the other hand, humified organic matter shows little relevance in this tree, and so it is inferred that this component is less important in the retention of this metal. Contributions by Fe and Cr oxides are not significant either, regarding both relevance and the changes to the tree structure or the relevance of the different variables, these components are not relevant [46, 32, 33].

As for the coefficients of importance for retention, it can be seen that the coefficient of importance of Mn oxides decreases regarding that of Fe or Al oxides in the adsorption process. In the case of clays, chlorite is the one showing the maximum value, while gibbsite is second in importance, shortly followed by kaolinite and vermiculite. In this case, as it was for adsorption, the percentage of sand is the most important component, so according to this model, the immobilization of this metal depends more on the oxides than it does on the clays. If working with coefficients of variation, the result applying main components would be the opposite [37] in the case that hematite was the most important component.

### Chromium

This metal is found naturally in some rocks, such as serpentinites, whose soils were sampled for this study and some previous studies [32, 33, 36, 37] where the regression tree model works similarly for adsorption and retention, with an *R*^*2*^ of 98.30% and 98.37%, respectively.

**Fig 3:**
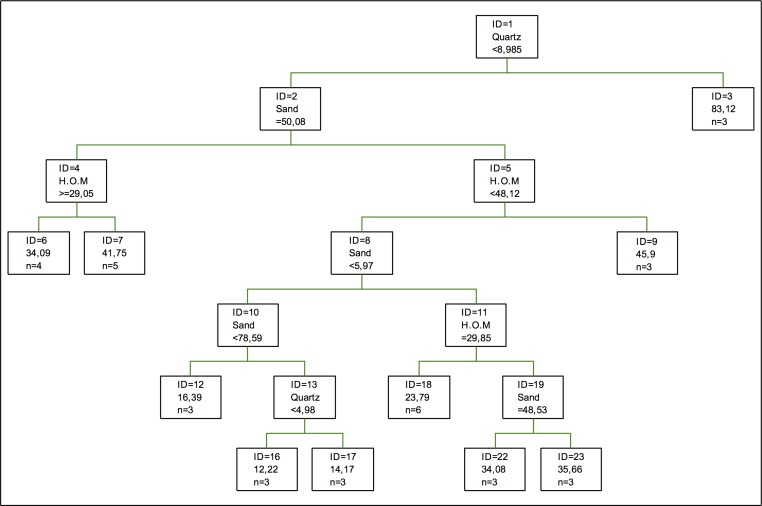
Regression tree for chromium adsorption. The inequalities < or ≤ correspond to the left branches and the opposite (> or ≥) correspond to the right branches.

In the modelling of chromium adsorption using regression tress, as shown in Fig 3, the first splitting is done according to the proportion of quartz in the soil, the second one being the percentage of sand. As for oxides, these do not appear in the optimal tree model even when Fe and Al oxides are considered as forming a complex, being taken as one only variable in the model, while clays are represented by kaolinite only, which also appears in [32, 33], so its role is more of a supporting role.

As for the coefficients of importance, the components with the highest values are quartz and humified organic matter, the conclusions by [47] not being that important, this being the most important variable, while Mn oxides show a much lower importance. In this model, the percentage of sand shows a lower importance than humified organic matter, so the adsorption of this metal depends less on the texture that it had been expected by other authors, although it coincides with the description by [32, 33].

Regarding the importance of clays, gibbsite is the most important one, followed by chlorite, kaolinite, and vermiculite. This protagonism of humified organic matter is due to the fact that these are the components which are coadjuvant to the adsorption action of quartz, given that this material is chemically inert [48].

**Fig 4:**
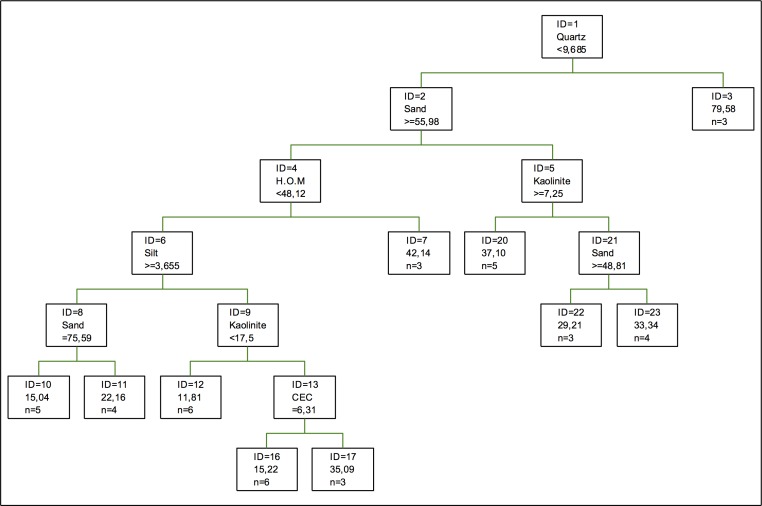
Regression tree for chromium retention. The inequalities < or ≤ correspond to the left branches and the opposite (> or ≥) correspond to the right branches.

Fig 4 shows the optimal tree for chromium retention following regression trees. In this case, oxides do not appear in the optimal explanatory model, but kaolinite does appear representing the clays. In the preceding analysis [32, 33], clays did not appear, but Fe oxides did. Nonetheless, now quartz is most explanatory, followed by the percentage of sand and humified organic matter, which partially matches the preceding model. The biggest difference between the model by [32] and the present model are the higher relevance of oxides (especially Fe oxides). The explanatory tree is simpler, given that the regression steps are few or that dramatic differences exist between one tree and the following [40].

In the analysis of the coefficients of importance for retention, a situation similar to that of the adsorption is observed, with quartz and the percentage of sand as the most important components. Humified organic matter loses importance, the importance of Mn oxides is null, and the importance of Fe and Al oxides is similar to that of the adsorption process, but less than gibbsite, which is the most importance clay, followed by chlorite, vermiculite and kaolinite. The importance of other components can also be seen, such as hematite, which, like M oxides, is not important for the retention of this metal. Complexes can be formed with different components, such as those with organic matter and clays [49].

### Copper

The regression tree model for this metal shows a goodness of fit of 99.33% for adsorption and 99.43% for retention.

**Fig 5:**
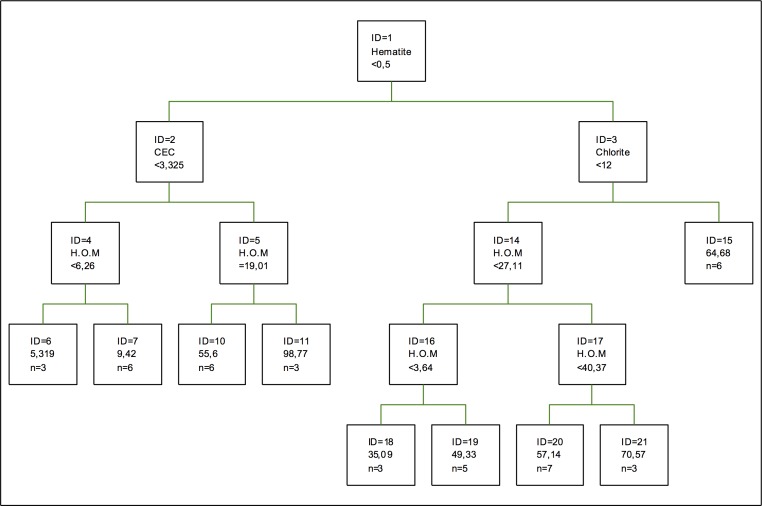
Regression tree for copper adsorption. The inequalities < or ≤ correspond to the left branches and the opposite (> or ≥) correspond to the right branches.

Fig 5 shows the optimal regression tree for copper adsorption. The component making the first splitting is the proportion of hematite, followed by the exchange capacity and chlorite. Clays do not appear and, in the case of oxides, Mn oxides do not appear either. Thus, copper adsorption shows a low dependency on the PH conditions and redox potential of the soil. Humified organic matter appears together with chlorite, so it can be said that this clay is modified by said component, in a situation similar to the one described in [49].

In this case, the most important component in copper adsorption is the percentage of sand, followed by humified organic matter and the proportion of quartz. As for clays, the most important one is kaolinite, followed by vermiculite, gibbsite and, lastly, kaolinite. As for oxides, Mn oxides are more important than Fe and Al oxides, these oxides show an adsorption curve or isotherm for copper of a hyperbolic type, its maximum concentration being much higher than that of other metals, such as nickel or zinc [50].

**Fig 6:**
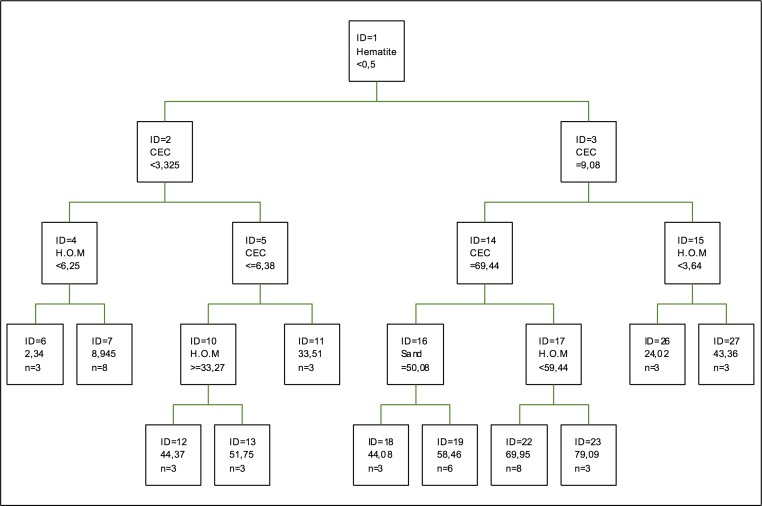
Regression tree for copper retention. The inequalities < or ≤ correspond to the left branches and the opposite (> or ≥) correspond to the right branches.

Fig 6 shows the regression tree for copper retention, being the variable that makes the first splitting the proportion of hematite, followed by cation-exchange capacity (CEC) and the proportion of humified organic matter. As for clays, they are not relevant in this case or for [33], updated by [51]. The same happens to amorphous oxides. Thus, retention is more dependent on the pH conditions and redox potential of the soil, so minerals are usually clays or hydrated, although their relevance is lower in the case of the union of two non-hydrated components. This can be modified when there are interactions with humified organic matter [52].

The highest coefficient of importance corresponds to CEC, followed by the percentage of sand and humified organic matter. As for clays, kaolinite is the most important one, followed by gibbsite, chlorite and, lastly, vermiculite, with the lowest coefficient of importance. In the case of Fe and Al oxides, they show a high affinity to this metal, although their importance in Galician soils is low, when they are found a structural complex adsorbing and retaining other types of metals, such as cobalt and lead [53].

### Nickel

For the adsorption and retention of nickel, the regression tree model works slightly better for the retention, with an *R*^*2*^ of 98.47%, while the goodness of fit for the adsorption process is 97.77% of the total variation.

**Fig 7:**
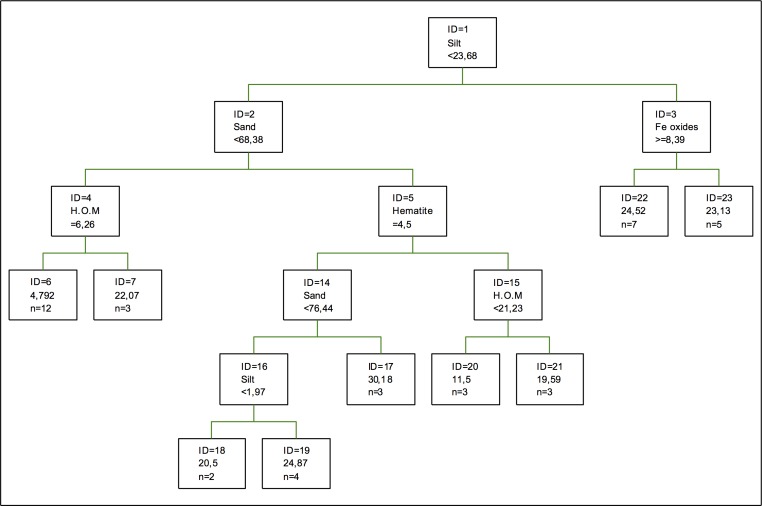
Regression tree for nickel adsorption. The inequalities < or ≤ correspond to the left branches and the opposite (> or ≥) correspond to the right branches.

The component found in the first splitting for nickel adsorption optimal regression tree (Fig 7) is the percentage of silt, followed by the percentage of sand and Fe oxides. Hematite is more relevant than it is in the model obtained by [32], where the formation of a structural complex between Fe and Al oxides is not considered and there Al oxides are relevant, being the only representative amorphous oxides. In the case of clays, they are not relevant for either model, which tells us that texture factors are more easily fitted to the nickel adsorption by the fitting method of regression trees [39].

Another way to perform the analyses is to consider the fraction or organic-mineral component rather than humified organic matter. In this case, the component in the same splitting does not vary, since it is still the percentage of silt but the tree shows less levels of splitting [46].

Regarding the coefficients of importance in this case it can be seen that the highest coefficient of importance is shown by the percentage of silt, followed by the proportions of humified organic matter and quartz. As for clays, gibbsite is the most important one, followed by kaolinite, vermiculite and chlorite. Oxides have a low importance, being a little higher for Mn oxides and it being possible to form complexes with several types of oxides [54] although clays can also be modified by oxides [52].

**Fig 8:**
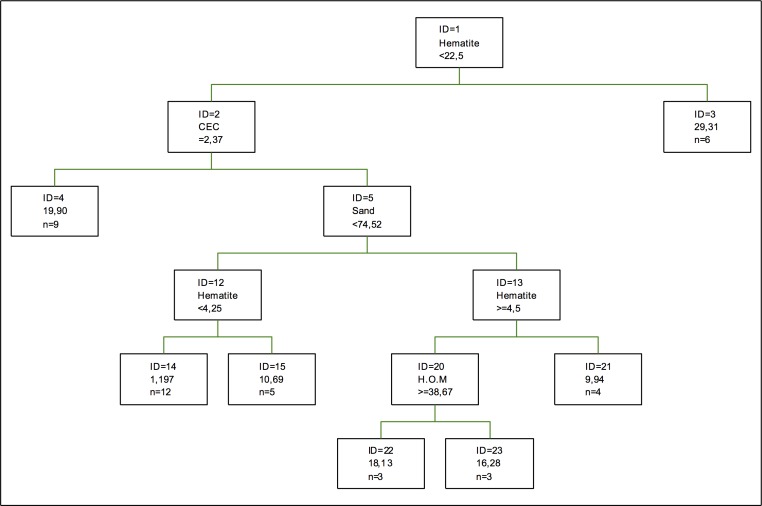
Regression tree for nickel retention. The inequalities < or ≤ correspond to the left branches and the opposite (> or ≥) correspond to the right branches.

Nickel retention tree can be seen in Fig 8. The first splitting is done according to the values of the proportion of hematite, followed by specific cation-exchange capacity and the percentage of sand. Clays do not appear, gibbsite being the only cay with certain relevance in the case of adsorption [32]. As for amorphous and non-hydrated oxides, they are not represented. The tree is completed with a splitting of humified organic matter, which tells us that nickel retention shows an action mechanism where hydrated components or components with a high capacity to adsorb water are preponderant, together with a certain texture. Therefore, a good soil with a good nickel retention would be a soil formed in a place with high rainfall or humidity in the atmosphere, not necessarily linked to an aquatic ecosystem [55].

Regarding the coefficients of importance, silt is the component with the highest coefficient of importance, followed by the proportion of mica and the percentage of sand. As for the importance of clays and oxides, their importance is not very high in general. The most important clay is kaolinite, while for the oxides the highest importance is for Fe oxides. Thus, this retention mainly occurs in mica and it is very dependent on the size of the grain rather than on the degree of hydration or the degree of crystallization of the different components [56].

### Lead

**Fig 9:**
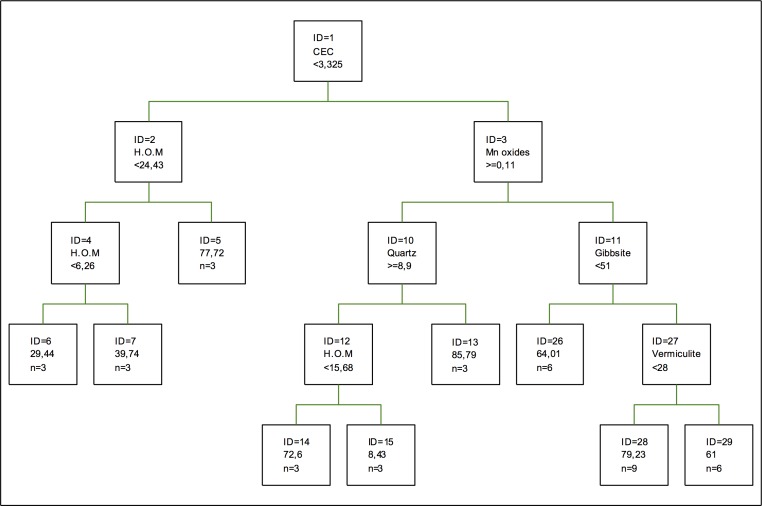
Optimal tree for lead adsorption. The inequalities < or ≤ correspond to the left branches and the opposite (> or ≥) correspond to the right branches.

**Fig 10:**
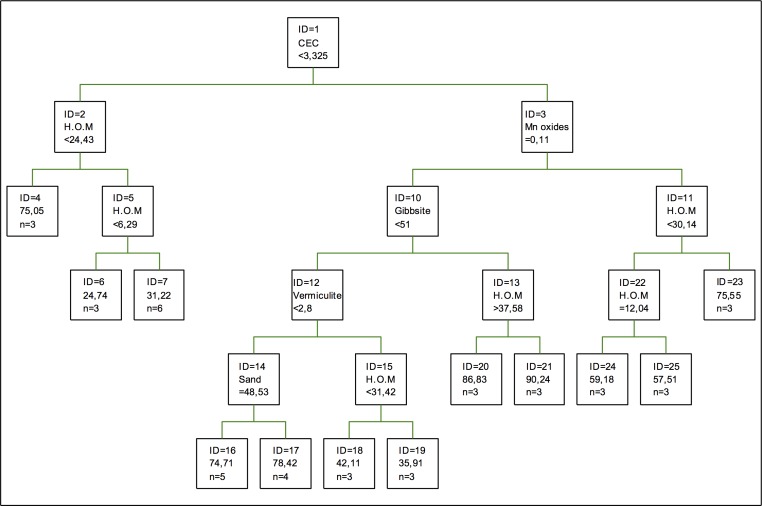
Optimal tree for lead retention. The inequalities < or ≤ correspond to the left branches and the opposite (> or ≥) correspond to the right branches.

The goodness of fit for lead is similar for adsorption (99.83%) and retention (99.76%). Fig 9 shows the regression tree for lead adsorption, whilst Fig 10 shows the same for lead retention. In the case of adsorption, the first splitting is done depending on the specific cation-exchange capacity, while humified organic matter and Mn oxides are found on a second level. As for clays, only vermiculite appears, although gibbsite, kaolinite and vermiculite appear in [32]. It is thus postulated that, in the adsorption of this metal, several types of components add their effects as if they were one only component [49].

Regarding the analysis of the coefficients of importance, humified organic matter has the second highest coefficient of importance, after the percentage of sand, and followed by chlorite and CEC. As for clays, chlorite is the most important one, a long distance from gibbsite, kaolinite and vermiculite, which is the least important clay. This adsorption depends on the pH conditions and redox potential of the soil, which determine the specific cation-exchange capacity [17]. Adsorption occurs more in clays than in oxides or humified organic matter. These differences in the adsorption can be explained by their different crystallographic structures [57].

In the retention process (Fig 10), the first splitting is also done by CEC, followed by humified organic matter (replacing the percentage of sand proposed in the model by [32] and Mn oxides, which are the only significant oxides. As for clays, vermiculite appears, but his process is less dependent on the proportion of these compounds than adsorption [48].

As for the coefficients of importance, the first three components are the same as in the case of adsorption, but the third position is for the percentage of silt rather than chlorite, even though the importance of the different oxides and clays is similar, and thus the same comment is applicable to both processes, in this case the importance of chlorite and humified organic matter cannot be said to be similar, and so they are not assumed to be found a complex as the one described in [49].

### Zinc

The regression model for zinc works better for the adsorption, with a goodness of fit of 93.60% of the total variance, while the percentage for adsorption falls to 88.48% of said variation.

**Fig 11:**
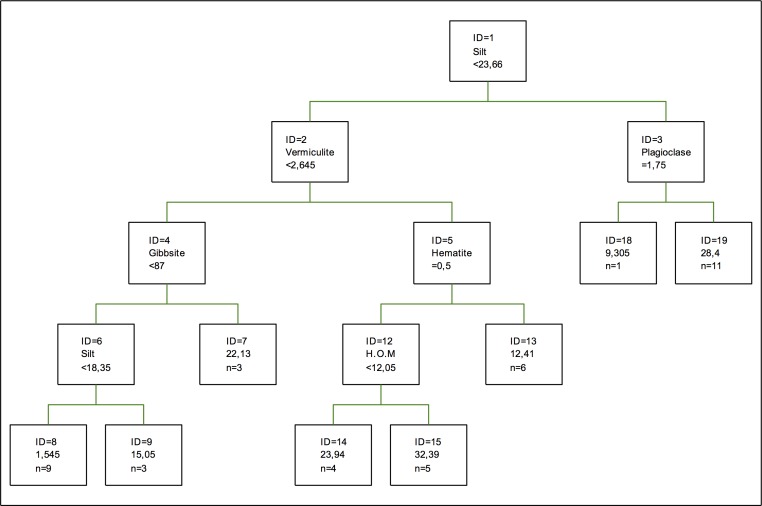
Optimal tree for zinc adsorption. The inequalities < or ≤ correspond to the left branches and the opposite (> or ≥) correspond to the right branches.

Zinc adsorption optimal tree is shown in Fig 11. The percentage of silt is the component making the first splitting, also appearing in later splittings. Vermiculite and plagioclase are at a second level. Gibbsite and vermiculite are the only clays represented. In the case of amorphous and non-hydrated oxides, they are not representative, so the adsorption of this metal is made preferentially in clays and hydrated metals such as hematite. In the case of clays, when coefficients of equilibrium are used, the adsorption shows a linear isotherm l [58].

As for the coefficients of importance, CEC is the most important variable for zinc adsorption, followed by quartz and chlorite, which is the most important clay, followed by gibbsite and vermiculite, kaolinite being the least important clay. In the case of oxides, Fe and Al oxides are most important than Mn oxides. In this model, the importance of the percentage of silt and chlorite is similar to that of quartz, which tells us that these three materials can form an adsorbent complex, just like Fe and Al oxides or, in some cases, Al and Mn oxides and hydroxides [54].

**Fig 12:**
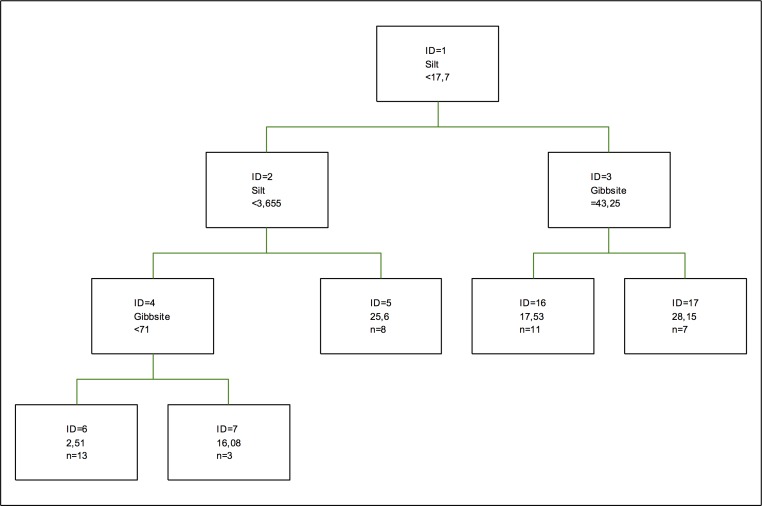
Optimal tree for zinc retention. The inequalities < or ≤ correspond to the left branches and the opposite (> or ≥) correspond to the right branches.

In the optimal zinc retention tree (Fig 12), only the percentage of silt (which is the one making the first splitting) and gibbsite are considered significant, so the model proposed in this tree is similar to those proposed by [32] and [46] using a competitive model. Thus, in the case of the behaviour of zinc in the soil, there are more influencing elements than those initially considered, as in the case when the competence between metals and the formation of the so-called organic-mineral complex are considered [46].

As for the coefficients of importance, quartz is the component with the highest importance, followed by the percentage of silt and the percentage of sand. Gibbsite is the most important clay, followed by chlorite, vermiculite and kaolinite in this order. Regarding the oxides, Fe and Al oxides show a higher coefficient of importance than Mn oxides. This situation is comparable to the one described in [59], where oxides are mixed with silicates, and also with the study by [47], where oxides show an amorphous structure, the aim of the study being to assess the behaviour of these oxides in the adsorption of chromium,

### Combined analysis of the importance of the variables

In comparison, oxides and clays show a similar importance in the adsorption and retention processes, except for certain variations. For adsorption, the results of the coefficients of the different components are shown in the Table 4.

**Table 4:** Coefficients of importance for the adsorption of the different metals.

	Cd ads	Cr ads	Cu ads	Ni ads	Pb ads	Zn ads
Al.oxides	2.085	2.230	0.527	0.858	0.538	1.223
CEC	6.460	4.674	9.743	2.645	5.957	2.806
Chlorite	0.896	4.766	1.724	0.769	6.053	2.200
Fe.oxides	2.598	2.944	0.527	0.858	0.538	1.843
Gibbsite	0.384	5.792	2.743	2.317	5.169	1.336
H.o.m.	5.757	5.963	12.222	3.321	6.158	1.296
Hematite	1.179	0.715	1.032	1.882	2.718	0.799
Kaolinite	0.714	2.083	4.919	1.419	3.929	0.338
Mica	3.325	3.213	3.540	2.357	0.774	1.358
Mn.oxides	1.599	1.086	1.655	1.918	0.825	0.000
Plagioclase	1.510	4.621	4.058	0.714	0.000	1.358
Quartz	3.790	8.144	11.766	3.174	4.943	2.409
Sand	7.403	5.874	12.740	2.243	7.118	1.409
Silt	4.687	7.291	10.548	3.803	5.208	2.006
Vermiculite	0.300	1.394	2.260	0.840	2.052	0.776

**H.O.M.** = Humified organic matter.

The following results are obtained related to the coefficients of importance, as shown in Table 4:

- Chlorite is the most important clay for the adsorption of cadmium, followed by kaolinite and gibbsite, vermiculite being the least important clay, although with much lower values than the most important components, such as the percentage of sand, CEC and humified organic matter, although in previous studies, such as [32], this variable was observed not to be among those which adsorb the most cadmium. As or oxides, Fe oxides are the most importance, with a value of the coefficient of importance similar to that of Al oxides but not equal, as found in [17].
- Gibbsite is the most important clay for the adsorption on chromium, followed by chlorite and kaolinite. As for gibbsite, this clay shows a coefficient of importance similar to that of humified organic matter [60], which is the third most important variable after the percentage of sand and the proportion of quartz. The importance of oxides are much lower, in contrasts with the results obtained by [32], where Fe and Me oxides are important for the adsorption of this metal.
- In the adsorption of copper, clays are of little importance compared to humified organic matter [61] and the percentage of sand. The most important clay is kaolinite. With a lower importance of oxides, copper can be adsorbed jointly with zinc [38].
- In the adsorption of nickel, gibbsite is the most relevant clay, coinciding with the description by [32], while Mn oxides are the most important oxides. This lower relationship between oxides and Ni adsorption has been observed by other authors, such as [46].
- Chlorite is the most important clay in the adsorption of lead, followed by gibbsite. As for oxides, Mn oxides are the most important, confirming the findings by [32], where these oxides, CEC and the composition of the clay fraction of the soil are the components which best explain the adsorption of this metal.
- In the adsorption of zinc, chlorite is the most important clay, followed by gibbsite. As for oxides, Fe oxides are the most important, while Mn oxides are not important for the adsorption of this metal. Co-adsorption with copper is not observed either, given that the variables explaining both processes are different [38].

As for the retention of the different metals, the results are shown in Table 5.

**Table 5:** Coefficients of importance for the retention of the different metals.

	Cd ret	Cr ret	Cu ret	Ni ret	Pb ret	Zn ret
Al.oxides	1.875	2.236	0.000	0.855	0.000	1.882
CEC	3.153	4.528	6.189	5.873	8.027	3.349
Chlorite	1.369	3.555	2.568	0.637	4.318	2.542
Fe.oxides	1.875	2.833	0.495	1.492	0.324	2.439
Gibbsite	1.359	3.818	3.351	1.982	3.354	2.872
H.o.m.	1.386	3.731	4.845	3.565	8.528	3.526
Hematite	1.937	0.000	1.882	1.966	1.927	0.855
Kaolinite	1.058	1.701	3.876	2.842	3.026	1.294
Mica	3.096	4.814	0.421	6.596	0.000	2.079
Mn.oxides	1.392	0.000	1.323	0.838	0.842	0.557
Plagioclase	1.402	4.012	0.000	3.309	1.511	1.897
Quartz	2.981	5.604	2.477	7.248	6.684	4.797
Sand	4.829	5.268	4.955	6.081	10.507	3.652
Silt	2.505	3.863	2.477	8.290	8.203	4.743
Vermiculite	0.485	2.733	1.684	1.025	1.873	1.351

**H.O.M.** = Humified organic matter.

In the retention of the metals, related to the importance of the variables (Table 5), it can be seen that:

- Just like for adsorption, chlorite is the most important clay in the retention of cadmium, having lower important values than the most important variables, such as the percentage of sand and CEC. As for oxides, Fe and Al oxides have a similar importance, which does not coincide with the description by [32], where clays show a higher importance and Mn oxides are the most relevant oxides.
- In the retention of chromium, just like in the case of adsorption, gibbsite and chlorite are the most important clays. In the case of oxides, Fe oxides are the most important, coinciding with the descriptions by [32] and [59].
- Kaolinite is the most important clay for copper retention, just like in the case of adsorption. As for oxides, they show a lower importance, and Mn oxides are not importance, so their affinity with copper is low, although they can be associated with clays for a higher efficiency [62].
- For nickel retention, kaolinite is the most important clay, followed by gibbsite. As for oxides, Fe oxides are the most important ones, although their importance is lower than the coefficient of kaolinite, which means clays predict the retention of this metal better than oxides [32].
- In lead retention, the most important clay is gibbsite. The contribution of oxides is not very important, although they are important for [32]. Hematite content is more important, also appearing in [46].
- In zinc retention, gibbsite is the most important clay, while Fe oxides are the most important oxides when these compounds are considered, although they do not appear in the optimal tree. This contribution by clays, together with the fact that the percentage of silt is the most important variable for Zn retention, coincides with the descriptions by [32, 46].

With these techniques, it is possible to study how it affects the different components of an ecosystem depending on its characteristics [63], where it is described how the use of multivariate techniques (not considered as *Machine Learning*) enables to know how the migration of inorganic pollution, both metallic and non-metallic, occurs.

From a broader perspective, these techniques enable us to know what the most serious contamination problems are and to select the best bioremediation method, if it is possible. This was the case with the selection of the best plant species for the restoration of the dump in a manganese mine, where different aspects, such as the concentration of heavy metals, were studied. Apart from manganese, cadmium, copper and zinc were mainly found. After studying the accumulation of heavy metals, it was found that the best species for revegetation were *Cynodon dactilon* and *Humulus scandens* [64].

## Conclusions

As general conclusions, the following can be mentioned:

Regression tree models (CART) allowed ranking the relative importance of the different soil components according to their relevance coefficients, on the adsorption and retention of 6 metals using 14 different soils. The cross-validation method allowed the improving of the models in terms of R^2^ in all metals retention and absorption, except in the case of Zn retention, in which there was no change.

The behaviour of the different adsorption and retention models for the 6 studied metals are different, with the first variable splitting the regression tree being quite variable.

The metals can be sorted out in two groups. One of them includes Cd, Ni and Zn, which generally show a high importance of the texture components such as the percentage of sand. The other group, including Cr, Cu and Pb, shows that the exchange capacity, humified organic matter and the proportion of hematite are the most important variables, as it happens in [32, 46].

Lastly, in some cases, there are cases of co-adsorption of several metals or of to components having a joint adsorption, as it happens with gibbsite in the adsorption of chromium [60]. As for the co-adsorption of different metals, the analysis does not detect the co-adsorption of copper and zinc proposed by [38].

## Supporting Information

S1 Table Original data from 42 soils. (PDF)

## References

[1] Becherucchi ME, Benavides H, Vallarino EA. Effect of Taxonomic Aggregation in Macroalgae Assemblages in a Rocky Shore of Mar del Plata, Argentina, Southwest Atlantic Ocean. Thalassas 2014; 30(1): 9–20

[2] Navarro–Aviñó JP, Aguilar–Alonso L, López-Moya JR. Aspectos bioquímicos y genéticos de la tolerancia y acumulación de metales pesados en plantas Ecosistemas 2007; (2) 1–17

[3] Kesavan K, Murugan A, Venkatesan V, Kumar BSV. Heavy Metal Accumulation in Molluscs and Sediment from Uppanar Estuary, Southeast Coast of India. Thalassas 2013; 29(2): 15–21

[4] Kesavan A, Raja P, Ravi V, Rajagopal S. Heavy Metals in *Thelescopium telescopium* and Sediments from two Stations of Vellar Estuary Southeast Coast of India. Thalassas 2010; 26(1): 35–41

[5] Cervantes C, Espino-Saldaña AE, Acevedo-Aguilar F, León-Rodríguez IL, Rivera-Cano ME, Ávila-Rodríguez M, et al Interacciones microbianas con metales pesados Microbiología ALAM 2006; 48(2) 209–218 17578093

[6] Nies DH. Efflux heavy metal resistance in prokaryotes FEMS Microbiology Reviews 2003; (27) 313–339 12829273 10.1016/S0168-6445(03)00048-2

[7] Ahmad–Ansuri ML, Aquil F. Biosorption of Ni, Cr and Cd by metal tolerant *Aspergillus niger* and *Penicilium* sp using simple and multi–metal solution Indian Journal of experimental Biology 2006; (44) 73–76 16430095

[8] Espino-Saldaña AF. Aislamiento y caracterización de hongos resistentes a cromato nativos de desechos industriales. Tesis de licenciatura RBE Facultad de química. Universidad de Guanajuato. 2002.

[9] Jasso-Chávez R, Moreno-Sánchez R. Cytosol- mitochondria transfer of reducing equivalents by a lactate shuttle in heterotrophic *Euglena* European Journal of Biochemistry 2003; (270) 4942–4951 14653820 10.1046/j.1432-1033.2003.03896.x

[10] Navarro M, Torres-Márquez MF, González-Moreno S, Devars S, Hernández R, Moreno-Sánchez R. Comparison of physiological changes in Euglena gracilis during exposition to heavy metals during autotrophic and heterotrophic cells Comparative biochemical physiology 1997; (116C) 265–272

[11] Pereira WG, De Sigüeira D, Martínez CA, Pulattini M. Gas Exchange and chlorophyll fluorescence in four cytrus rootstocks under aluminium stress. Journal of plant physiology 2002; (157) 513–520.

[12] Susplugas S, Srivastaba A, Strasser RJ Changes in the photosynthetic activities during several stages of vegetative growth of *Spirodela prholyiza*; Effect of chromate. Journal of Plant Physiology 2002; (157) 503–512.

[13] Marmiroli M, Antonioli G, Maestri M, Marmiroli N. Evidence of involvement of plant lingo-celulosic structure in the sequestration of Pb; An X–Ray spectroscopy analysis Environmental Pollution 2005; (134) 217–227 10.1016/j.envpol.2004.08.004 15589649

[14] Mendoza-Cozati D, Moreno-Sánchez R. Control of glutatyone and phytochelatin under cadmium stress. Pathway modeling for plants Journal of Theoretical Biology 2006; (238) 919–936 16125728 10.1016/j.jtbi.2005.07.003

[15] Arazi T, Sunkar R, Kaplan B, From H. A tobacco plasma membrane calmodulin binding–transporter confers Ni^2+^ tolerance and Pb^2+^ hypersensitivity in transgenic plants Plant Journal 1999; (20) 161–182 10.1046/j.1365-313x.1999.00588.x 10571877

[16] Ezaki B, Gardner RC, Ezaki Y, Matsumoto H. Expression of aluminium–induced genes in transgenic *Arabidopsis;* Plants can ameloidate aluminium stress and/or oxidative stress Plant Physiology 2000; (122) 657–666 10712528 10.1104/pp.122.3.657 PMC58900

[17] Covelo EF. Adsorción y desorción competitiva de metales pesados en suelos, PhD Thesis. Universidad de Vigo, Spain. 2005.

[18] Gought LP, Shaklette HT, Case AA. Element concentrations toxic to plants, animals and man U.S. Department of interior Geological Survey Geological Survey 1979; Bulletin 1466 Washington CD

[19] Connell Does W. Basic concepts in environmental chemistry Lewis Publishers NY USA 1997.

[20] Hussain D, Haidon MJ, Wangy, Wong E, Sherson SM, Camakaris J, et al P–type ATPase heavy metal transporters with roles in essential zinc metabolism in *Arabidopsis* The plant cell 2004; (16) 1327–1339 15100400 10.1105/tpc.020487 PMC423219

[21] Singh A, Prasad SM. Remediation of heavy metal contaminated ecosystem: an overview on technology advancement. Int. J. Environ. Sci. Technol. 2015; 12:353–366.

[22] Vanthime M, Maes A (2007) The removal heavy metals from dredged sediments to mechanical Denver filtration, the contribution of the filtration and extractment Land Contamination and reduction 15(1) 15–30

[23] Shang Shihh S, Wang Kia S, Ua Chung Y, Chan Chih Y, Chon Chug T. Remediation of metal contaminated soil by an integrated soil electrolysis process Soil and Sediment Contamination 2015; 14(6) 559–569

[24] Adeleye AS, Conway JR, Garner K, Huang YX, Su YM, Keller AA. Engineered nanomaterials for water treatment and remediation. Costs, benefits, and applicability. Chemical Engineering Journal 2016; 286:640–662.

[25] Aguilar J, Dorronsoro C, Fernández E, Fernández J, García I, Sierra M, et al Remediation of As–contaminated soils in the Guadiamar river basin (SW Spain) Water, Air and Soil pollution 2007; (180) 109–118.

[26] Tejada M, Hernández MT and García C (2007) Application of two organic wastes in a soil polluted by lead, effects on the soil enzymatic activities Journal of Experimental Chemistry 66(1) 226–235 10.2134/jeq2006.0252RA 17215229

[27] Dilks RT, Monette F, Glaus M. The major parameters on biomass pyrolysis for hyperaccumulatiove plants. A review. Chemosphere 2016; 146: 385–395. doi: 10.1016/j.chemosphere.2015.12.062 26741543 10.1016/j.chemosphere.2015.12.062

[28] Méndez MO, Maier RM. Phytoremediation of mine tailings in temperate and arid environments Reviews of Environmental Science and Biotechnology 2008; (7) 47–59

[29] Yadav R, Arora P, Kumar S, Chaudury A. Perspectives for genetic engineering of poplars for enhanced phytoremediation abilities. Ecotoxicology 2010; 19(8): 1574–1588. doi: 10.1007/s10646-010-0543-7 20848189 10.1007/s10646-010-0543-7

[30] Turek M, Korolevicz T, Oibe J. Removal of heavy metals from sewage sludge and soil fertilizers Soil and Sediment Contamination 2005; 14(2) 143–154

[31] Le Countre DT. A meta-Analysis and Risk-Assessment of Heavy metals Intake in Common Garden Vegetables, PhD Thesis, Tennessee University. 2001.

[32] Covelo EF, Vega FA, Andrade ML. Sorption and desorption of Cd, Cr, Cu, Ni, Pb and Zn by a fibric histosol and its organo-mineral fraction Journal of Hazardous Materials 2008; (159) 342–347 10.1016/j.jhazmat.2008.02.025 18384955

[33] Covelo EF, Matías JM, Reigosa MJ, Andrade ML. A tree regression of factors determining the sorption and retention of heavy metals by soil Geoderma 2008; (147) 75–85

[34] World reference basis for soil resources. ISSS ISRIC FAO. Roma. 1999.

[35] Guitián F, Carballas T. Técnicas de análisis de suelos. Editorial Pico Sacro. Santiago de Compostela. 1976.

[36] Covelo EF, Vega FA, Andrade ML. Simultaneous sorption and desorption of Cd, Cr, Cu, Ni, Pb and Zn in acid soils, 1 Selectivity sequences Journal of Hazardous Materials 2007; (147) 852–861 10.1016/j.jhazmat.2007.01.123 17346879

[37] Covelo EF, Vega FA, Andrade ML. Simultaneous sorption and desorption of Cd, Cr, Cu, Ni, Pb and Zn in acid soils II Soil ranking and influence of soil characteristics Journal of Hazardous Materials 2007; (147) 862–870 10.1016/j.jhazmat.2007.01.108 17350755

[38] Covelo EF, Vega FA, Andrade ML. Competitive sorption and desorption of heavy metals by individual soil components Journal of Hazardous Materials 2007; (148) 308–315 10.1016/j.jhazmat.2006.09.018 17049729

[39] Breiman L, Friedman J, Olshen A, Stone C. Classification and regression tress Wadsworth. 1984.

[40] Hastie T, Tibshirani R, Friedman J. Data Mining, Inference and Prediction Stanford. Springer–Verlag New York 2001.

[41] HastieE T, Tibshirani R. Generalized Aditive Models ed Chapman and Hall London 1990.

[42] The R Project. 2011. Package ‘rpart’. http://cran.r-project.org/web/packages/rpart/rpart.pdf

[43] Therneau TM, Atkinson EJ. An Introduction to Recursive splitting Using the RPART Routines. Mayo Foundation 2011.

[44] Covelo EF, Vega FA, Andrade ML. Heavy metal adsorption by humic umbrisols; Selectivity sequences and competitive sorption kinetics Journal of Colloid and Interface Science 2004; (281) 1–8 10.1016/j.jcis.2004.07.024 15476767

[45] Mohapatra M, Anand S. Studies on sorption of Cd(II) on Tata Chromite mine overburden Journal of Hazardous Materials 2007; (148) 553–559 10.1016/j.jhazmat.2007.03.008 17416465

[46] Vega FA, Matías JM, Andrade ML, Reigosa MJ, Covelo EF. Classification and regression trees (CARTs) for modelling the sorption and retention of heavy metals by soil Journal of Hazardous Materials 2008; (13) 615–624 10.1016/j.jhazmat.2009.01.016 19200658

[47] Auso EA, Sánchez AG, Querol X. Adsorption of Cr(VI) from synthetic solutions and electroplating wastewaters an amorphous aluminium oxides Journal of Hazardous Materials 2007; (142) 191–198 10.1016/j.jhazmat.2006.08.004 16978771

[48] Sipos P, Nemet T, Kovacskis M, Mohai I. Sorption of copper zinc and lead on soil mineral phases Chemosphere 2008; (73) 463–469 10.1016/j.chemosphere.2008.06.046 18674797

[49] Chaturvedi PK, Seth CS, Mista V. Selectivity sequences and sorption capacities of phosphatidic clay and humus with soil towards the heavy metals present in zinc mine tailings Journal of hazardous materials 2008; (147) 698–705 10.1016/j.jhazmat.2007.01.064 17303325

[50] McKenzie RM. The sorption of heavy metals by the lower oxides of manganese Geoderma 1971; (8) 29–35

[51] Vega FA, Andrade ML, Covelo EF. Influence of soil properties on the sorption and retention of cadmium, copper and lead, separately and together, by 20 soil horizons: Comparison of linear regression and tree regression analyses, Journal of Hazardous Materials 2010; (174) 1–3, 522–533 10.1016/j.jhazmat.2009.09.083 19811872

[52] Elem E. Removal of copper ions by modified Unye clay, Turkey Journal of Hazardous Materials 2008; (150) 235–244 10.1016/j.jhazmat.2008.02.035 18375056

[53] Dong D, Liu L, Hua X, Lu Y. Comparison of lead, cadmium, copper and cobalt adsorption onto metal oxides and organic materials in natural surface coatings Microchemical Journal 2007; (85) 270–275

[54] Park M, Lyeal Chon C, Jin Seo Y, Kar Yeo S, Choi J, Konarmeni S, et al Reactions of Cu^2+^ and Pb^2+^ with Mg/Al layered double hydroxide Applied Clay Science 2007; (37) 143–148

[55] Bi X, Feng X, Yang Y, Li X, Sin GPY, Qiu G, et al Heavy metals in an impacted wetland system; a typical case of southwestern China Science of the Total Environment 2007; (387) 257–268 17822743 10.1016/j.scitotenv.2007.07.059

[56] Fontes MPF, De Matos AT, Da Costa LM, Neves JCL. Competitive adsorption of zinc, cadmium, copper and lead in three highly–weathered Brazilian soils Communications of Soil Science and Plant Analysis 2000; (31) 2939–2958

[57] Gascova OL, Bukaty MB. Sorption of different cations onto clays; Modelling approach of ion exchange and surface complexation Physics and Chemistry of the Earth 2008; (33) 1050–1055

[58] Nelly S, Alyüz B. Adsorption of copper and zinc from aqueous solutions onto natural clay Journal of Hazardous materials 2007; (149) 226–233 10.1016/j.jhazmat.2007.04.109 17560022

[59] Yuhua W, Ye H, Yehua L. Adsorption mechanisms of Cr(VI) of the bauxite tailings Minerals engineering 2008; (21) 913–917

[60] Bloomfield C. *in* Greenlan DJ and HayesS MHR (eds) The translocation of metals in soils en The Chemistry of Soil Processes John Willey & sons Chichester 1981.

[61] Lindsai WL. *in* Alloway BJ (ed) Heavy metals in soils Blackie Academic & Professional London 1995.

[62] Boonfueng T, Axe L, Xu Y. Properties and structure of manganese oxide–coated clay Journal of Colloid and Interface Science 2005; (281) 80–92 10.1016/j.jcis.2004.08.048 15567383

[63] Zeng C, Wun Luo Y, Zang H, Christian P. Identifying sources of soil inorganic pollutants on a regional scale using of a multivariate statistical approach; Role of pollutant migration and soil physicochemical properties Environmental Pollution 2008; (208) 470–476 17604890 10.1016/j.envpol.2007.04.017

[64] Wang X, Liu Y, Zeng G, Chai L, Xian X Song X, et al Pedological characteristics of Mn mine tailings and metal accumulation by native plants Chemosphere 2008; (72) 1260–1266. 10.1016/j.chemosphere.2008.05.001 18555510

