# Supplementary material for: Analysis of the Importance of Oxides and Clays in Cd, Cr, Cu, Ni, Pb and Zn Adsorption and Retention with Regression Trees
Source: PLoS One. 2017 Jan 10;12(1):e0168523. doi: 10.1371/journal.pone.0168523 (PMC5224874; doi:10.1371/journal.pone.0168523)
Supplement: S1 Table — (PDF) [file pone.0168523.s001.pdf]

soil number	HOM (mg kg^{-1})	phH2O	sand (%)	Silt (%)	clay (%)	SCEC	quartz	plagioclase	Mica
1	82.95	4.7	74.11	2.87	23.02	4.61	1.5	1.5	0.35
2	88.48	4.71	75.01	3.77	21.22	4.68	1.67	2	0.48
3	88.48	4.69	73.21	1.67	25.12	4.82	1.33	1	0.22
4	69.98	4.5	49.53	32.25	18.22	4.6	1.18	1.5	0.27
5	59.22	4.51	48.43	32.45	19.12	4.86	1.34	2	0.38
6	53.83	4.49	48.63	31.35	20.02	4.9	1.04	1	0.17
7	27.03	4.9	65.06	20.08	14.86	2.61	0.97	1.5	0.22
8	43.24	4.91	65.96	20.98	13.06	2.65	1.1	2	0.32
9	21.62	4.89	64.16	19.18	16.66	2.59	0.84	1	0.14
10	100.52	5.1	51.52	33.66	14.82	2.04	7.56	1	0.15
11	100.52	5.11	52.42	34.56	13.02	2.13	8.33	1.5	0.24
12	96.33	5.09	50.62	32.76	16.62	2.15	6.82	0.5	0.07
13	5.3	5.00	60.43	17.97	21.6	1.02	4.39	0	0
14	5.3	5.01	60.33	17.87	21.8	1.13	5.02	0	0
15	5.3	4.99	59.53	19.07	21.4	1.09	3.79	0	0
16	97.83	4.6	47	26.34	26.66	5.91	1.05	0	0
17	81.52	4.61	44.63	28.24	27.13	6.1	1.2	0	0
18	89.67	4.59	46.19	26.44	27.37	6.27	0.92	0	0
19	21.43	4.51	71.89	1.82	26.29	0.36	8.54	0	0
20	16.07	4.52	70.79	2.02	27.19	0.4	9.38	0	0
21	21.43	4.5	70.99	1.92	27.09	0.36	7.74	0	0
22	42.44	5.1	77.06	3.54	19.4	4.24	7.76	7	1.36
23	50.93	5.11	77.26	3.44	19.3	4.84	8.53	7.5	1.52
24	42.44	5.09	77.16	3.34	19.5	4.18	7.03	6.5	1.2
25	40.34	6.3	65.55	5.24	29.21	5.67	4.38	7	2.04
26	42.44	6.31	65.45	5.14	29.41	6.16	5.12	7,5	2,26
27	42.44	6.29	65.65	5.34	29.01	5.56	3.68	6.5	1.84
28	15.96	6.6	82.2	8.29	9.51	10.07	3.3	7	0.66
29	15.96	6.61	82.1	8.19	9.71	9.82	3.82	7.5	0.77
30	7.98	6.59	82.17	8.3	9.53	9.82	2.81	6.5	0.55
31	125.36	5.4	44.83	17.42	37.75	4.1	10.89	0	0
32	150.43	5.41	44.73	17.32	37.95	4	11.93	0	0
33	175.5	5.39	44.93	17.52	37.55	4.1	9.87	0	0
34	15.8	6.4	26.81	34.31	38.88	10.92	4.7	0	0
35	14.22	6.41	26.71	34.21	39.08	11.22	5.3	0	0
36	13.43	6.39	26.91	34.41	38.68	10.38	4.13	0	0
37	123.27	4.4	75.82	10.75	13.43	8.28	2.93	0	0
38	126.43	4.41	75.02	10.65	14.33	8.34	3.18	0	0
39	126.43	4.39	74.92	9.85	15.23	8.28	2.69	0	0
40	71.12	4.9	60.5	6.97	32.53	7.92	9	0	2
41	71.12	4.91	60.4	6.9	32.7	7.93	9.5	0	2.5
42	86.92	4.89	60.6	6.5	32.9	7.94	8.5	0	1.5

Kaolinite	Vermiculite	Gibbsite	Chlorite	hematite	amorphous	Mn oxides	Fe oxides	Al oxides	Cdads	Cdret
20	4.6	32	7.37	20	4.6	0.28	12.63	16.68	6.54	4.00
22	5.26	34	8.13	22	5.26	0.25	11.44	15.11	6.69	5.61
18	3.98	30	6.64	18	3.98	0.29	13.07	17.27	6.62	4.74
0	0	45	8.2	25	4.56	0.03	8.55	11.29	30.05	25.70
0	0	47	8.99	27	5,16	0.03	8.08	10.68	37.42	34.03
0	0	43	7.45	23	3.98	0.03	8.23	10.87	34.01	29.86
1,5	0.22	45.5	6.76	0	0	0.04	8.67	11.46	2.96	0.00
2	0.32	47.5	7.49	0	0	0.03	8.59	11.35	2.51	0.00
1	0.14	43.5	6.07	0	0	0.04	9.07	11.98	2.17	0.00
12	1.78	12	1.78	12	1.78	0.13	13.21	17.44	28.61	24.79
14	2.2	14	2.2	14	2.2	0.11	11.72	15.48	27.60	24.05
10	1.39	10	1.39	10	1.39	0.11	11.59	15.31	28.10	24.50
20	4.39	60	13.16	0	0	0	2.86	3.78	25.96	23.59
22	5.02	62	14.16	0	0	0	2.69	3.55	27.44	25.05
18	3.79	58	12.2	0	0	0	3.14	4.15	24.41	21.92
31	5.03	31.5	5.11	31	5.03	0	7.21	9.52	36.77	32.49
33	5.65	33.5	5.74	33	5.65	0.01	8.09	10.68	37.73	33.34
29	4.45	29.5	4.52	29	4.45	0	7.63	10.07	35.97	31.43
13	3.42	13	3.42	0	0	0	10.22	13.49	26.36	22.08
15	4.08	15	4.08	0	0	0	11.34	14.97	28.17	23.78
11	2.79	11	2.79	0	0	0	10.76	14.21	24.58	20.03
20	3.88	20	3.88	0	0	0.06	13.65	18.03	35.07	29.97
22	4.47	22	4.47	0	0	0.07	13.18	17.41	35.49	30.55
18	3.33	18	3.33	0	0	0.06	12.07	15.94	33.86	28.67
15	4.38	55	16.06	1.5	0.44	0.03	20.59	27.19	24.15	11.78
17	5.12	57	17.16	2	0.6	0.03	27.17	35.89	24.02	12.71
13	3.68	53	15	1	0.28	0.03	24.07	31.79	23.02	11.41
20	1.89	30	2.83	7	0.66	0.03	3.79	5	13.14	4.91
22	2.27	32	3.31	7.5	0.77	0.03	3.47	4.59	18.44	8.26
18	1.54	28	2.39	6.5	0.55	0.03	3.55	4.69	13.44	2.99
0	0	47	18.27	0	0	0	2.53	3.34	5.45	3.88
0	0	49	19.49	0	0	0.01	2.47	3.26	8.93	7.37
0	0	45	17.09	0	0	0.01	2.54	3.36	7.27	5.63
7	0.94	51	6.85	7	0.94	1.41	68.77	90.83	20.26	5.97
7.5	1.07	53	7.59	7.5	1.07	1.3	59.66	78.8	20.43	7.05
6.5	0.81	49	6.14	6.5	0.81	1.52	74.36	98.21	19.91	6.00
2	0.65	87	28.3	2	0.65	0.01	5.43	7.17	36.40	27.20
2.5	0.84	89	29.75	2.5	0.84	0.01	5.55	7.33	36.00	27.68
1.5	0.47	85	26.89	1.5	0.47	0.01	4.63	6.12	37.22	27.05
87	2	0	0	0	3	0.18	9.76	12.89	5.26	0.00
89	2.5	0	0	0	3	0.16	9.2	12.15	3.48	0.00
85	1.5	0	0	0	3	0.15	8.84	11.67	6.09	0.00
